# Near-Complete Genome Sequence of a Swine Norovirus GII.11 Strain Detected in Japan in 2018

**DOI:** 10.1128/MRA.00014-20

**Published:** 2020-04-23

**Authors:** Ayaka Okada, Yasuo Inoshima

**Affiliations:** a Laboratory of Food and Environmental Hygiene, Cooperative Department of Veterinary Medicine, Faculty of Applied Biological Sciences, Gifu University, Gifu, Japan; b Education and Research Center for Food Animal Health, Gifu University, Gifu, Japan; c Joint Graduate School of Veterinary Sciences, Gifu University, Gifu, Japan; d United Graduate School of Veterinary Sciences, Gifu University, Gifu, Japan; KU Leuven

## Abstract

Here, we report the near-complete genome sequence of swine norovirus strain SwNoV/Sw1/2018/JP. The genome was genetically similar (90.2%) to that of the only other swine norovirus strain previously detected in Japan (SW/NV/swine43/JP). In conclusion, genome sequences of swine noroviruses in Japan have not been changed significantly in the past 15 years.

## ANNOUNCEMENT

Noroviruses (NoVs) belong to the genus *Norovirus* in the family *Caliciviridae* and are classified into seven genogroups (GI to GVII), based on the phylogenetic analysis of the capsid gene (1). Swine NoVs (SwNoVs) belong to the GII genogroup and were first reported in the Shizuoka Prefecture in Japan in 1997 (2). Although SwNoVs have been detected worldwide (3–6), only three complete viral genome sequences can be found in GenBank. Here, we used next-generation sequencing to determine the near-complete genome sequence of an SwNoV strain detected in Japan. Furthermore, the novel sequence was compared with that of a previously detected strain from Japan, which was available in GenBank.

Viral RNA of strain SwNoV/Sw1/2018/JP was extracted from a fecal sample collected from a healthy pig in Gifu Prefecture, Japan, in 2018. The supernatant of the fecal suspension (10% [wt/vol]) was filtered and concentrated by ultracentrifugation (155,000 × *g* for 2 h at 4°C), and the pellet was resuspended in sterile phosphate-buffered saline. RNA was extracted from the suspension using a viral RNA minikit (Qiagen, Hilden, Germany) and stored at –80°C until use. The presence of SwNoV was investigated by nested PCR, as described previously (7). RNA quantity and purity were assessed using a NanoDrop 2000c spectrophotometer (Thermo Fisher Scientific, Waltham, MA, USA) and a 2100 bioanalyzer (Agilent Technologies, Santa Clara, CA, USA), respectively. Prior to library preparation, rRNA was removed using the Ribo-Zero rRNA removal kit for bacteria (Epicentre, Madison, WI, USA). High-throughput RNA transcriptome sequencing (RNA-seq) libraries were prepared with the TruSeq stranded mRNA sample preparation kit (Illumina, San Diego, CA, USA) according to the manufacturer’s instructions. RNA paired-end sequencing (2 × 101 bp) was performed on an Illumina HiSeq 2500 sequencing system (Illumina), and data were analyzed by the Hokkaido System Science Co., Ltd. (Sapporo, Japan). In total, 46,017,338 reads were generated. Adapters and low-quality reads were removed using Trimmomatic v0.32 (8) using a sliding-window approach with a minimum Phred quality score of 33. Processed and cleaned reads were mapped to the SwNoV reference genome, SW/NV/swine43/JP (GenBank accession number AB126320), using Bowtie 2. After filtering and trimming, 45,699,506 reads remained, 1,743 of which were mapped to the SwNoV reference genome, and Illumina read alignments were visually inspected using IGV v2.4.5 (9). The final average coverage was 23-fold. PCRs and Sanger sequencing were performed to examine parts of the SwNoV genome that had not been determined by RNA-seq. Regions at 1 to 537, 756 to 1255, 1307 to 1957, 2092 to 2615, 3142 to 3756, 3735 to 4317, 5064 to 7325, and 7325 to 7537 nucleotides (nt), corresponding to the SW/NV/swine43/JP genome sequence, were amplified using eight sets of PCR primers, which were designed based on the draft genome sequence of SwNoV/Sw1/2018/JP. For amplification of the 5′ and 3′ ends, we used the rapid amplification of cDNA ends (RACE) strategy with some modifications. In brief, short adaptor sequences (5′-amino linker C_6_ [AmC_6_]-TCGTATGCCGTCT-phosphate [PHO]-3′ for the 5′ end and 5′-PHO-TCGTATGCCGTCT-AmC_7_-3′ for the 3′ end), were ligated to the genome of SwNoV/Sw1/2018/JP by T4 RNA ligase 1 (New England Biolabs, Ipswich, MA, USA). Ligated DNA-RNA hybrids were reverse transcribed and amplified using the primers for the adaptors and the gene-specific primers for SwNoV/Sw1/2018/JP. The amplicons were subsequently analyzed by Sanger sequencing in a Prism 3130 genetic analyzer with the BigDye Terminator v3.1 cycle sequencing kit (Applied Biosystems, Foster City, CA, USA). Sanger reads and the contigs generated from the Illumina reads were merged manually using SnapGene v4.1.9 software (GSL Biotech LLC, Chicago, IL, USA). PCR of the 5′ end was unsuccessful and, although we observed clearly the electropherogram ending that connected with the adapter for the 3′ end, 447 and 14 nt from the 5′ and 3′ ends, respectively, were missing when the genome was aligned with the SW/NV/swine43/JP genome. Consistent with our result, some studies reported variability in the 3′ untranslated regions in some RNA viruses (10, 11). The near-complete genome sequence of the SwNoV/Sw1/2018/JP strain, consisting of 7,090 nt, with a G+C content of 52.6%, was obtained.

**Fig. 1:**
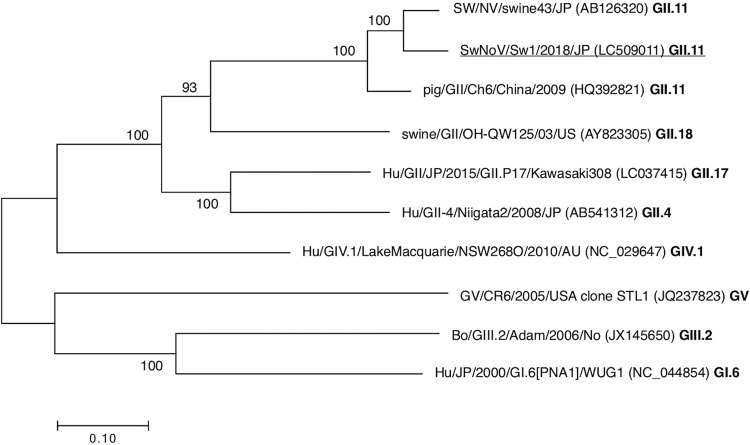
Phylogenetic tree of the near-complete genome sequences of NoV strains. The phylogenetic tree was constructed using the maximum likelihood method in MEGA7 (12), which provided statistical support by bootstrapping with 1,000 replicates. The GenBank accession numbers of the representative sequences are shown in parentheses. The genogroups and genotypes of these strains are shown in bold. The strain detected in this study is underlined. Bootstrap values of >80% (1,000 replicates) are shown on the nodes. Bar, number of substitutions per site.

The sequence of the SwNoV/Sw1/2018/JP genome was aligned with the previously reported NoV complete genomes using ClustalW. A phylogenetic tree was constructed and revealed that SwNoV/Sw1/2018/JP belonged to the same cluster as SW/NV/swine43/JP (Fig. 1). Comparative analysis at the nucleotide level showed that the genome sequence of SwNoV/Sw1/2018/JP was 90.2% identical to that of SW/NV/swine43/JP.

To our knowledge, this is the first report to compare the near-complete genome sequences of two SwNoVs detected in the same country. Our data suggest that the SwNoV genome has not significantly changed in the past 15 years. Additional full-genome sequencing of other SwNoVs detected worldwide is required to elucidate how SwNoVs circulate among pig populations.

### Data availability.

The genome nucleotide sequence has been deposited in GenBank under accession number LC509011. This paper describes the first version, LC509011.1. The raw sequence data from BioProject PRJDB9141 were submitted to the DDBJ Sequence Read Archive (DRA)/SRA under experiment accession number DRX193326.
